# Supplementary material for: Human Exposure to Pesticides in Dust from Two Agricultural Sites in South Africa
Source: Toxics. 2022 Oct 21;10(10):629. doi: 10.3390/toxics10100629 (PMC9610731; doi:10.3390/toxics10100629)
Supplement: Supplementary file 1 [file toxics-10-00629-s001.zip › toxics-1920543-supplementary.pdf]

Supplementary Information

Human Exposure to Pesticides in Dust from Two Agricultural Sites in South Africa

Céline Degrendele [1,2,*], Roman Prokeš [1,3], Petr Šenk [1], Simona Rozárka Jílková [1], Jiří Kohoutek [1], Lisa Melymuk [1], Petra Přibylová [1], Mohamed Aqiel Dalvie [4], Martin Röösli [5,6], Jana Klánová [1], and Samuel Fuhrimann [5,6,7]

[1] RECETOX, Faculty of Science, Masaryk University, 625 00 Brno, Czech Republic
[2] Aix-Marseille University, CNRS, LCE, 13003 Marseille, France
[3] Global Change Research Institute of the Czech Academy of Sciences, 603 00 Brno, Czech Republic
[4] Centre for Environmental and Occupational Health Research, School of Public Health and Family Medicine, University of Cape Town, Cape Town 7925, South Africa
[5] University of Basel, 4002 Basel, Switzerland
[6] Swiss Tropical and Public Health Institute (Swiss TPH), 4002 Basel, Switzerland
[7] Institute for Risk Assessment Sciences (IRAS), Utrecht University, 3584 Utrecht, The Netherlands
* Correspondence: celine.degrendele@recetox.muni.cz

Number of pages: 17

Number of Tables: 9

Number of Figures: 7

TABLE OF CONTENTS

List of Tables:

Table S1: Average recoveries and their standard deviations determined from spiking experiments.... S2
Table S2: Input parameters used for the assessment of daily intakes from dust ingestion S3

Table S3: Basic statistics of pesticide levels found in all dust samples. S4
Table S4: Basic statistics of pesticide levels found in all household dust samples collected at Hex River Valley. S5
Table S5: Basic statistics of pesticide levels found in all household dust samples collected at Grabouw. S6
Table S6: Daily intake (in pg kg^{-1} day^{-1}) of individual pesticides via dust ingestion for a child S7
Table S7: Daily intake (in pg kg^{-1} day^{-1}) of individual pesticides via dust ingestion for an adult S8
Table S8: Hazard quotients of children due to the exposure to individual pesticides via dust ingestion, inhalation, and soil ingestion found in this study S9
Table S9: Hazard quotients of adults due to the exposure to individual pesticides via dust ingestion, inhalation, and soil ingestion found in this study S10

List of Figures:

Figure S1: Dust levels of individual pesticides in 12 households and one school measured at day 1 and day 7 .... S11
Figure S2: Boxplots of concentrations of individual pesticides in dust samples S12
Figure S3: Contribution of three exposure pathways on the daily uptake of pesticides of a children living at farm and village locations at Hex River Valley and Grabouw using the maximum concentrations.. S13
Figure S4: Contribution of three exposure pathways on the daily uptake of pesticides of a children living at farm and village locations at Hex River Valley and Grabouw using the median concentrations and high ingestion rate S14
Figure S5: Contribution of three exposure pathways on the daily uptake of pesticides of an adult living at farm and village locations at Hex River Valley and Grabouw using the median concentrations ... S15
Figure S6: Contribution of three exposure pathways on the daily uptake of pesticides of an adult living at farm and village locations at Hex River Valley and Grabouw using the maximum concentrations S16
Figure S7: Contribution of three exposure pathways on the daily uptake of pesticides of an adult living at farm and village locations at Hex River Valley and Grabouw using the median concentrations and the high ingestion rate of dust S17

Table S1: Average recoveries and their standard deviations determined from spiking experiments. (n3)

	Dust
Acetochlor	42.5 (2.9)
Alachlor	96.1 (13.6)
Atrazine	60.9 (10.7)
Azinphos methyl	113.8 (27.5)
Carbaryl	69.9 (3.0)
Carbendazim	109.8 (10.3)

Chlorotoluron	80.6 (4.1)
Chlorpyrifos	55.3 (14.8)
Chlorsulfuron	120.1 (1.9)
Diazinon	49.6 (7.2)
Dimethachlor	95.8 (6.4)
Dimethoate	90.2 (4.2)
Diuron	85.9 (3.5)
Fenitrothion	96.8 (10.8)
Fluroxypyr	110.7 (17.5)
Isoproturon	75.2 (3.7)
Malathion	109.7 (20.3)
Metamitron	95.6 (5.2)
Metazachlor	96.5 (4.0)
Metribuzin	89.4 (4.7)
Parathion methyl	100.4 (3.7)
Pendimethalin	87.7 (9.4)
Pirimicarb	48.0 (10.3)
Prochloraz	95.4 (1.3)
Propiconazole	110.6 (3.2)
Pyrazon	89.8 (2.0)
Simazine	85.8 (5.2)
S-metolachlor	84.7 (4.2)
Tebuconazole	106.7 (6.5)
Terbuthylazine	94.6 (2.9)

Table S2: Input parameters used for the assessment of daily intakes from dust ingestion.

		Children	Adult	Reference
$IngR_{dust}$	Median	0.04	0.02	[75]
	High	0.1	0.06	[75]
AF		0.14	0.14	[76]
BW		31.8	80	[75]

Table S3: Basic statistics of pesticide levels found in all dust samples. QF indicates quantification frequency in percentage; IQR indicates interquartile range. For propiconazole, S-metolachlor, diuron, and malathion, only the concentrations above the quantification limits were considered, while for the remaining pesticides, imputed data were considered for statistics.

		**chlorpyrifos**	**terbuthylazine**	**carbaryl**	**diazinon**	**carbendazim**	**tebuconazole**	**propiconazole**	**S-metolachlor**	**diuron**	**malathion**
All samples n=54	QF	96	91	74	74	61	59	9	4	4	4
	Mean	1,180	9.57	960	119	21.6	14.2				
	Median	288	4.75	247	10.9	7.02	4.77				
	Min	0	0.05	5	0.29	0.14	0.19	3.63	10.3	26.8	43.9
	Max	19,500	90.8	17,200	2,210	279	99.0	12.5	46.6	26.8	150
	IQR25	143	2.97	20.0	1.32	0.15	0.20				
	IQR75	968	9.51	544	31.2	15.3	16.1				
Households n=50	QF	96	90	76	72	60	58	8	4	2	4
	Mean	1,250	9.30	1,020	122	16.3	13.6				
	Median	365	4.54	247	9.29	7.02	4.38				
	Min	0	0.05	5	0.29	0.14	0.19	3.63	10.3	26.0	43.9
	Max	19,500	90.8	17,200	2,210	257	99.0	12.5	46.6	26.8	150
	IQR25	135	2.76	64.0	0.30	0.15	0.20				
	IQR75	986	9.25	544	31.2	14.2	15.8				

Table S4: Basic statistics of pesticide levels (in ng g^{-1}) found in all household dust samples collected at Hex River Valley. QF indicates quantification frequency in percentage; IQR indicates interquartile range. For propiconazole, S-metolachlor, diuron, and malathion, only the concentrations above the quantification limits were considered, while for the remaining pesticides, imputed data were considered for statistics.

		**chlorpyrifos**	**terbuthylazine**	**carbaryl**	**diazinon**	**carbendazim**	**tebuconazole**	**propiconazole**	**S-metolachlor**	**diuron**	**malathion**
Hex River Valley (farm, village, day 1, day 7) n=25	QF	96	96	60	68	36	92	8	0	0	0
	Mean	1,810	5.38	292	136	17.5	25.1				
	Median	398	4.47	102	6.41	0.15	15.7				
	Min	0	0.05	5	0.29	0.14	0.20	3.63			
	Max	19,500	11.0	1,980	1,680	257	99.0	9.60			
	IQR25	142	3.04	5	0.29	0.15	7.96				
	IQR75	1,850	7.70	266	15.5	7.45	35.4				
Hex River Valley (farm, village, day 7) n=19	QF	95	95	58	63	37	100	11	0	0	0
	Mean	2,040	5.26	292	105	21.7	30.6				
	Median	255	4.47	82	7.71	0.15	24.1				
	Min	0	0.05	5	0.29	0.14	2.98	3.63			
	Max	19,500	11.0	1,980	1,680	257	99.0	9.60			
	IQR25	126	2.70	5	0.29	0.15	10.0				
	IQR75	1,880	7.68	247	24.2	7.26	38.4				
Hex River Valley (village, day 7) n=9	QF	89	89	67	78	33	100	22	0	0	0
	Mean	2,520	4.41	485	209	5.29	18.6				
	Median	189	3.87	82	15.3	0.15	10.6				
	Min	0	0.05	5	0.29	0.14	2.98	3.63			
	Max	19,500	10.5	1,980	1,680	32.2	63.5	9.60			
	IQR25	127	2.26	5	6.41	0.14	4.43				
	IQR75	425	6.30	283	54.5	7.06	24.1				
Hex River Valley (farm, day 7) n=10	QF	100	100	50	50	40	100	0	0	0	0
	Mean	1,610	6.03	118	9.75	36.4	41.4				
	Median	333	6.14	54	2.38	0.15	35.9				
	Min	73	1.65	5	0.29	0.14	7.96				
	Max	4,900	11.0	521	36.1	257	99.0				
	IQR25	152	3.47	5	0.29	0.15	19.0				
	IQR75	3,100	8.13	166	13.0	25.6	59.5				

Table S5: Basic statistics of pesticide levels (in ng g^{-1}) found in all household dust samples collected at Grabouw. QF indicates quantification frequency in percentage; IQR indicates interquartile range. For propiconazole, S-metolachlor, diuron, and malathion, only the concentrations above the quantification limits were considered, while for the remaining pesticides, imputed data were considered for statistics.

		**chlorpyrifos**	**terbuthylazine**	**carbaryl**	**diazinon**	**carbendazim**	**tebuconazole**	**propiconazole**	**S-metolachlor**	**diuron**	**malathion**
Grabouw (farm, village, day 1, day 7) n=25	QF	96	84	92	76	84	24	8	8	4	8
	Mean	690	13.2	1,740	109	15.1	2.04				
	Median	268	4.62	525	11.2	11.0	0.20				
	Min	0	0.05	5	0.29	0.14	0.19	3.63	10.3	26.8	43.9
	Max	4,700	90.8	17,200	2,210	64.1	19.60	12.5	46.6	26.8	150
	IQR25	133	2.70	207	4.37	5.94	0.19				
	IQR75	948	13.6	1,660	37.4	17.8	0.20				
Grabouw (farm, village, day 7) n=19	QF	95	84	100	68	84	32	11	5	5	11
	Mean	801	14.5	1,820	137	14.2	2.62				
	Median	366	4.62	537	10.9	11.0	0.20				
	Min	0	0.05	69	0.29	0.14	0.19	7.00			43.9
	Max	4,700	90.8	17,200	2,210	34.9	19.60	12.5	46.6	26.8	150
	IQR25	123	2.82	261	0.29	7.59	0.19				
	IQR75	961	13.5	1,320	39.2	22.1	1.45				
Grabouw (village, day 7) n=9	QF	89	89	100	89	100	44	22	11	11	11
	Mean	216	5.95	2,430	281	20.8	5.04				
	Median	113	4.62	540	40.9	14.3	0.20				
	Min	0	0.05	69	0.29	8.19	0.19	3.63			
	Max	1,070	13.6	17,200	2,210	34.9	19.60	12.5	46.6	26.8	43.9
	IQR25	86	2.94	207	11.19	10.1	0.20				
	IQR75	181	7.75	610	73.7	32.4	8.76				
Grabouw (farm, day 7) n=10	QF	100	80	100	50	70	20	0	0	0	10
	Mean	1,330	22.3	1,280	7.84	8.32	0.44				
	Median	714	7.53	475	2.55	7.59	0.19				
	Min	268	0.05	122	0.29	0.14	0.19				
	Max	4,700	90.8	4,180	37.4	30.2	2.60				150
	IQR25	422	2.95	328	0.29	0.17	0.19				
	IQR75	1,070	30.2	1,490	9.67	12.3	0.20				

Table S6: Daily intake (in pg kg^{-1} day^{-1}) of individual pesticides via dust ingestion for a child.

				**chlorpyrifos**	**terbuthylazine**	**carbaryl**	**diazinon**	**carbendazim**	**tebuconazole**	**propiconazole**	**S-metolachlor**	**diuron**	**malathion**
Median IngR	Hex River Valley	Village	Median	30.9	0.88	10.4	2.24	0.33	3.52	0	0	0	0
			Max	2,410	1.70	244	208	4.28	10.1	1.18	0	0	0
		Farm	Median	48.6	1.16	6.90	0.65	0.33	6.65	0	0	0	0
			Max	611	1.75	64.5	4.80	32.0	14.4	0	0	0	0
	Grabouw	Village	Median	26.4	1.12	94.9	6.05	10.9	0.16	0.26	0	0.71	0
			Max	146	2.52	2,150	273	19.0	2.69	2.05	5.74	4.72	5.41
		Farm	Median	100	1.48	86.9	1.32	10.1	0.16	0.26	0	0.71	0
			Max	594	12.0	546	5.97	18.5	0.59	0.51	0	1.42	18.4
Max IngR	Hex River Valley	Village	Median	77.3	2.20	26.0	5.60	0.82	8.81	0	0	0	0
			Max	6,037	4.24	611	519	10.7	25.1	2.96	0	0	0
		Farm	Median	122	2.90	17.3	1.61	0.82	16.6	0	0	0	0
			Max	1,530	4.39	161	12.0	79.9	36.1	0	0	0	0
	Grabouw	Village	Median	66.0	2.79	237	15.1	27.4	0.41	0.64	0	1.77	0
			Max	365	6.31	5,380	684	47.6	6.71	5.13	14.4	11.8	13.5
		Farm	Median	251	3.69	217	3.31	25.3	0.41	0.64	0	1.77	0
			Max	1,490	30.1	1,370	14.9	46.2	1.47	1.28	0	3.54	46.1

Table S7: Daily intake (in pg kg^{-1} day^{-1}) of individual pesticides via dust ingestion for an adult.

				**chlorpyrifos**	**terbuthylazine**	**carbaryl**	**diazinon**	**carbendazim**	**tebuconazole**	**propiconazole**	**S-metolachlor**	**diuron**	**malathion**
Median IngR	Hex River Valley	Village	Median	6.63	0.14	2.88	0.54	0.01	0.37	0	0	0	0
			Max	683	0.37	69.3	58.9	1.13	2.22	0.34	0	0	0
		Farm	Median	11.7	0.21	1.88	0.08	0.01	1.26	0	0	0	0
			Max	172	0.38	18.2	1.26	8.99	3.47	0	0	0	0
	Grabouw	Village	Median	3.97	0.16	18.9	1.43	0.50	0.01	0	0	0	0
			Max	37.4	0.48	602	77.3	1.22	0.69	0.44	1.63	0.94	1.54
		Farm	Median	25.0	0.26	16.6	0.09	0.27	0.01	0	0	0	0
			Max	165	3.18	146	1.31	1.06	0.09	0	0	0	5.23
Max IngR	Hex River Valley	Village	Median	19.9	0.41	8.63	1.61	0.02	1.11	0	0	0	0
			Max	2,050	1.10	208	177	3.38	6.67	1.01	0	0	0
		Farm	Median	35.0	0.64	5.64	0.25	0.02	3.77	0	0	0	0
			Max	515	1.15	54.7	3.79	27.0	10.40	0	0	0	0
	Grabouw	Village	Median	11.9	0.48	56.7	4.29	1.50	0.02	0	0	0	0
			Max	112	1.43	1,810	232	3.66	2.06	1.31	4.89	2.81	4.61
		Farm	Median	75.0	0.79	49.9	0.27	0.80	0.02	0	0	0	0
			Max	494	9.53	439.1	3.93	3.17	0.27	0	0	0	15.7

Table S8: Hazard quotients of children due to the exposure to individual pesticides via dust ingestion, inhalation, and soil ingestion found in this study. H, G, V, F, and IngR denote Hex River Valley, Grabouw, village, farm, and dust ingestion rate, respectively.

		chlorpyrifos	terbuthylazine	carbaryl	diazinon	carbendazim	tebuconazole	propiconazole	metolachlor	diuron	malathion	acetochlor	alachlor	atrazine	azinphos methyl	dimethachlor	isoproturon	metazachlor	pirimicarb	simazine
Children Cmedian (with Median IngR)	H_V	1.66E-04	1.24E-04	1.45E-05	1.32E-05	2.21E-07	2.77E-05	3.85E-08	2.60E-07	0.00E+00	4.63E-06	0.00E+00	6.80E-08	2.82E-08	0.00E+00	6.61E-08	6.43E-11	2.63E-09	5.71E-10	1.52E-06
	H_F	1.84E-04	1.24E-04	1.40E-05	5.20E-06	2.21E-07	2.78E-05	3.85E-08	2.60E-07	0.00E+00	4.63E-06	0.00E+00	6.80E-08	2.82E-08	0.00E+00	6.61E-08	2.60E-10	2.63E-09	5.71E-10	1.52E-06
	G_V	1.47E-04	1.74E-05	3.84E-04	3.22E-05	5.58E-07	5.62E-08	1.74E-08	2.37E-07	2.36E-07	4.04E-07	2.58E-06	6.80E-08	3.55E-09	4.60E-08	2.88E-08	6.36E-11	0.00E+00	0.00E+00	3.00E-07
	G_F	2.21E-04	1.75E-05	3.83E-04	8.57E-06	5.17E-07	5.62E-08	1.74E-08	2.37E-07	2.36E-07	4.04E-07	2.58E-06	6.80E-08	3.55E-09	4.60E-08	2.88E-08	6.36E-08	0.00E+00	0.00E+00	3.00E-07
Children Cmax (with Median IngR)	H_V	2.60E-03	1.83E-04	1.15E-04	1.04E-03	7.18E-07	2.80E-04	8.68E-08	3.98E-07	0.00E+00	9.53E-06	0.00E+00	1.04E-06	8.15E-08	0.00E+00	2.02E-07	1.33E-09	1.13E-08	2.00E-09	1.83E-06
	H_F	7.97E-04	1.83E-04	9.13E-05	2.85E-05	2.10E-06	2.80E-04	5.73E-08	3.98E-07	0.00E+00	9.53E-06	0.00E+00	1.04E-06	8.15E-08	0.00E+00	2.02E-07	1.33E-09	1.13E-08	2.00E-09	1.83E-06
	G_V	3.98E-04	3.34E-05	1.55E-03	1.38E-03	1.16E-06	1.26E-06	8.45E-08	7.03E-07	1.57E-06	6.68E-07	1.40E-05	5.20E-07	4.00E-08	1.20E-06	1.82E-07	6.52E-11	0.00E+00	0.00E+00	1.76E-06
	G_F	8.46E-04	3.58E-05	1.33E-03	4.04E-05	1.13E-06	1.19E-06	4.61E-08	6.45E-07	4.72E-07	1.10E-06	1.40E-05	5.20E-07	4.00E-08	1.20E-06	1.82E-07	6.52E-11	0.00E+00	0.00E+00	1.76E-06
Children Cmedian (with High IngR)	H_V	2.13E-04	1.24E-04	1.65E-05	2.99E-05	2.46E-07	2.79E-05	3.85E-08	2.60E-07	0.00E+00	4.63E-06	0.00E+00	6.80E-08	2.82E-08	0.00E+00	6.61E-08	2.60E-10	2.63E-09	5.71E-10	1.52E-06
	H_F	2.57E-04	1.24E-04	1.54E-05	1.00E-05	2.46E-07	2.81E-05	3.85E-08	2.60E-07	0.00E+00	4.63E-06	0.00E+00	6.80E-08	2.82E-08	0.00E+00	6.61E-08	2.60E-10	2.63E-09	5.71E-10	1.52E-06
	G_V	1.87E-04	1.78E-05	4.03E-04	7.76E-05	1.38E-06	6.44E-08	2.70E-08	2.37E-07	5.90E-07	4.04E-07	2.58E-06	6.80E-08	3.55E-09	4.60E-08	2.88E-08	6.36E-08	0.00E+00	0.00E+00	3.00E-07
	G_F	3.72E-04	1.80E-05	4.00E-04	1.85E-05	1.28E-06	6.44E-08	2.70E-08	2.37E-07	5.90E-07	4.04E-07	2.58E-06	6.80E-08	3.55E-09	4.60E-08	2.88E-08	6.36E-08	0.00E+00	0.00E+00	3.00E-07

Table S9 : Hazard quotients of adults due to the exposure to individual pesticides via dust ingestion, inhalation and soil ingestion found in this study. H, G, V, F and IngR denote Hex River Valley, Grabouw, village, farm and dust ingestion rate, respectively.

		chlorpyrifos	terbuthylazine	carbaryl	diazinon	carbendazim	tebuconazole	propiconazole	metolachlor	diuron	malathion	acetochlor	alachlor	atrazine	azinphos methyl	dimethachlor	isoproturon	metazachlor	pirimicarb	simazine
Adult Cmedian (with High IngR)	H_V	8.65E-05	6.14E-05	7.65E-06	8.99E-06	1.03E-07	1.37E-05	1.90E-08	1.29E-07	0.00E+00	2.30E-06	0.00E+00	3.40E-08	1.40E-08	0.00E+00	3.28E-08	8.53E-12	1.25E-09	7.14E-11	7.58E-07
	H_F	1.02E-04	6.14E-05	7.25E-06	2.20E-06	1.03E-07	1.38E-05	1.90E-08	1.29E-07	0.00E+00	2.30E-06	0.00E+00	3.40E-08	1.40E-08	0.00E+00	3.28E-08	8.53E-12	1.25E-09	7.14E-11	7.58E-07
	G_V	5.58E-05	8.62E-06	1.92E-04	2.24E-05	7.99E-08	2.60E-08	5.50E-09	1.18E-07	0.00E+00	2.01E-07	1.28E-06	3.40E-08	1.51E-09	2.40E-08	2.88E-08	8.40E-12	0.00E+00	0.00E+00	1.47E-07
	G_F	1.19E-04	8.69E-06	1.91E-04	2.29E-06	4.48E-08	2.60E-08	5.50E-09	1.18E-07	0.00E+00	2.01E-07	1.28E-06	3.40E-08	1.51E-09	2.40E-08	2.88E-08	8.40E-12	0.00E+00	0.00E+00	1.47E-07
Adult Cmedian (with Median IngR)	H_V	7.33E-05	6.13E-05	6.88E-06	3.63E-06	1.02E-07	1.37E-05	1.90E-08	1.29E-07	0.00E+00	2.30E-06	0.00E+00	3.40E-08	1.40E-08	0.00E+00	3.28E-08	8.53E-12	1.25E-09	7.14E-11	7.58E-07
	H_F	7.83E-05	6.13E-05	6.75E-06	1.37E-06	1.02E-07	1.37E-05	1.90E-08	1.29E-07	0.00E+00	2.30E-06	0.00E+00	3.40E-08	1.40E-08	0.00E+00	3.28E-08	8.53E-12	1.25E-09	7.14E-11	7.58E-07
	G_V	4.78E-05	8.54E-06	1.87E-04	8.11E-06	3.00E-08	2.56E-08	5.50E-09	1.18E-07	0.00E+00	2.01E-07	1.28E-06	3.40E-08	1.51E-09	2.40E-08	2.88E-08	8.40E-12	0.00E+00	0.00E+00	1.47E-07
	G_F	6.88E-05	8.56E-06	1.87E-04	1.40E-06	1.83E-08	2.56E-08	5.50E-09	1.18E-07	0.00E+00	2.01E-07	1.28E-06	3.40E-08	1.51E-09	2.40E-08	2.88E-08	8.40E-12	0.00E+00	0.00E+00	1.47E-07
Adult Cmax (with Median IngR)	H_V	7.75E-04	9.08E-05	5.03E-05	2.97E-04	3.07E-07	1.39E-04	3.69E-08	1.98E-07	0.00E+00	4.73E-06	0.00E+00	5.18E-07	4.01E-08	0.00E+00	1.00E-07	1.99E-10	5.63E-09	2.86E-10	9.02E-07
	H_F	2.63E-04	9.08E-05	4.35E-05	8.53E-06	7.00E-07	1.39E-04	2.85E-08	1.98E-07	0.00E+00	4.73E-06	0.00E+00	5.18E-07	4.01E-08	0.00E+00	1.00E-07	1.99E-10	5.63E-09	2.86E-10	9.02E-07
	G_V	1.41E-04	1.64E-05	7.06E-04	3.92E-04	1.65E-07	6.05E-07	2.74E-08	3.37E-07	3.13E-07	2.94E-07	6.97E-06	2.58E-07	1.96E-08	5.94E-07	9.06E-08	8.67E-12	0.00E+00	0.00E+00	8.68E-07
	G_F	2.68E-04	1.70E-05	6.45E-04	1.18E-05	1.57E-07	5.85E-07	1.65E-08	3.21E-07	0.00E+00	4.17E-07	6.97E-06	2.58E-07	1.96E-08	5.94E-07	9.06E-08	8.67E-12	0.00E+00	0.00E+00	8.68E-07

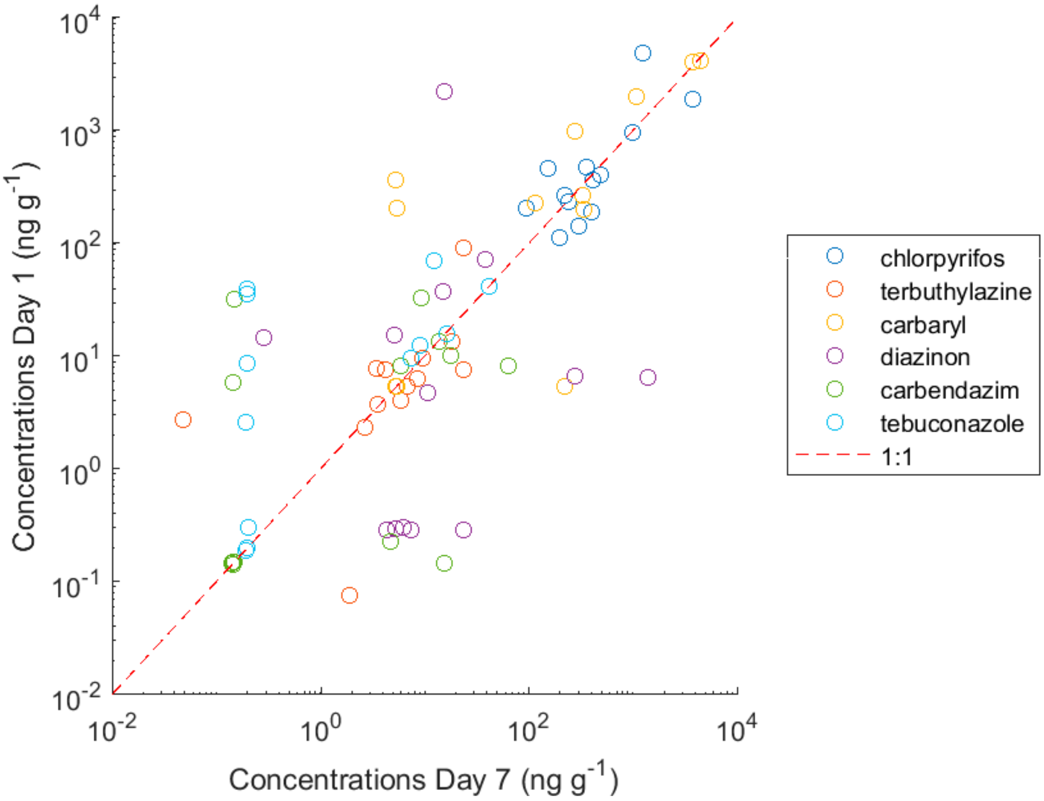

Figure S1: Dust levels of individual pesticides (in ng g^{-1}) in 12 households and one school measured at day 1 and day 7.

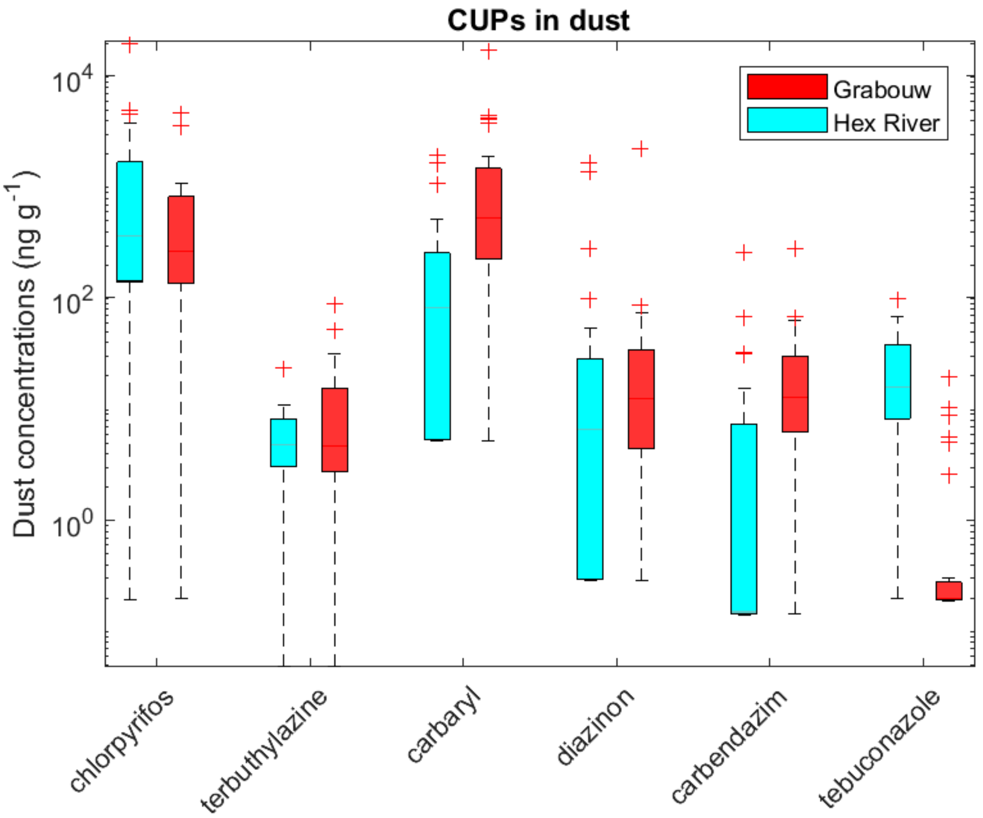

Figure S2: Boxplots of concentrations of individual pesticides in dust samples (in ng g^{-1}). All samples (i.e., day 1, day 7, village, farm, and schools, n=54) were considered. Boxplots represent the 25-75th percentile, whiskers represent the minimum and maximum values (excluding outliers which are shown as the red crosses) and the line within the box represents the median value.

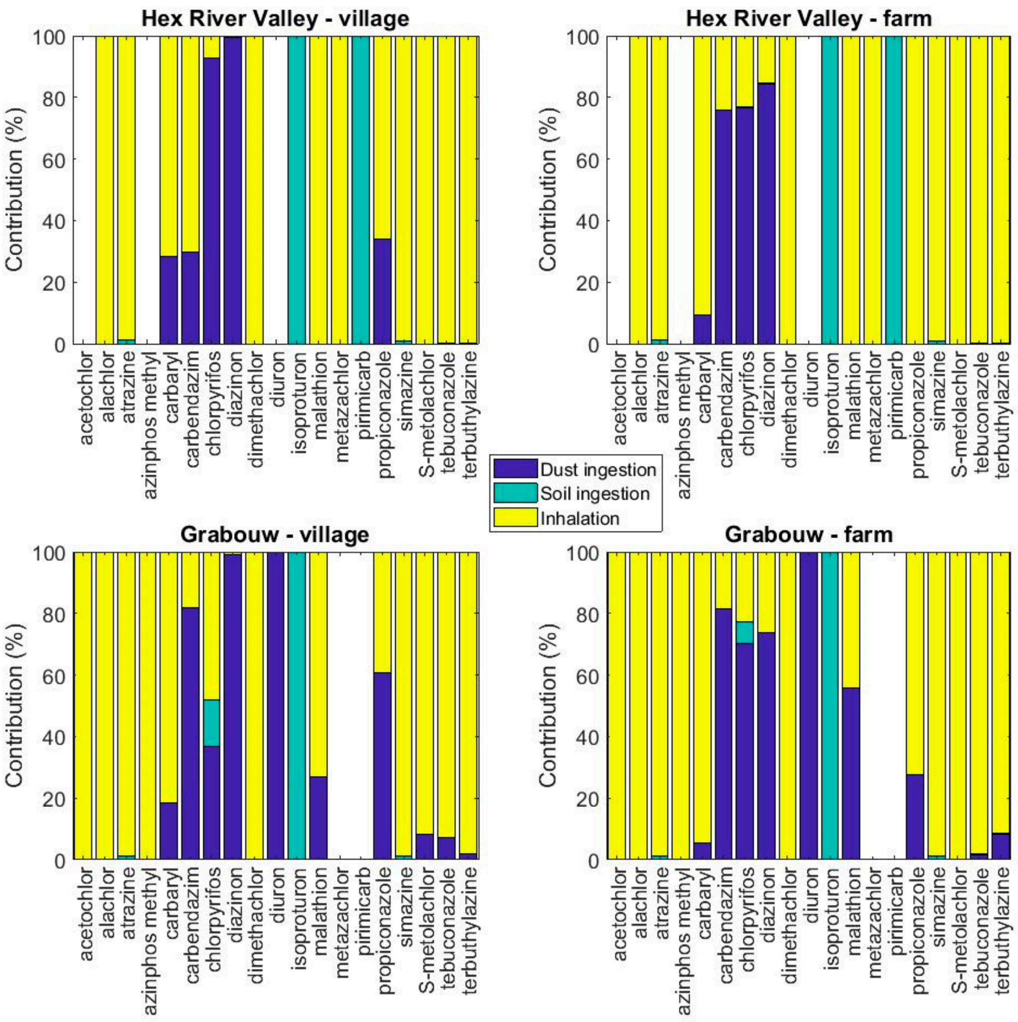

Figure S3: Contribution (in %) of three exposure pathways (dust ingestion, soil ingestion, and inhalation) on the daily uptake of pesticides of a children living at farm and village locations at Hex River Valley and Grabouw using the maximum concentrations. Data on the daily intakes from inhalation and soil ingestion of individual pesticides were obtained from [68].

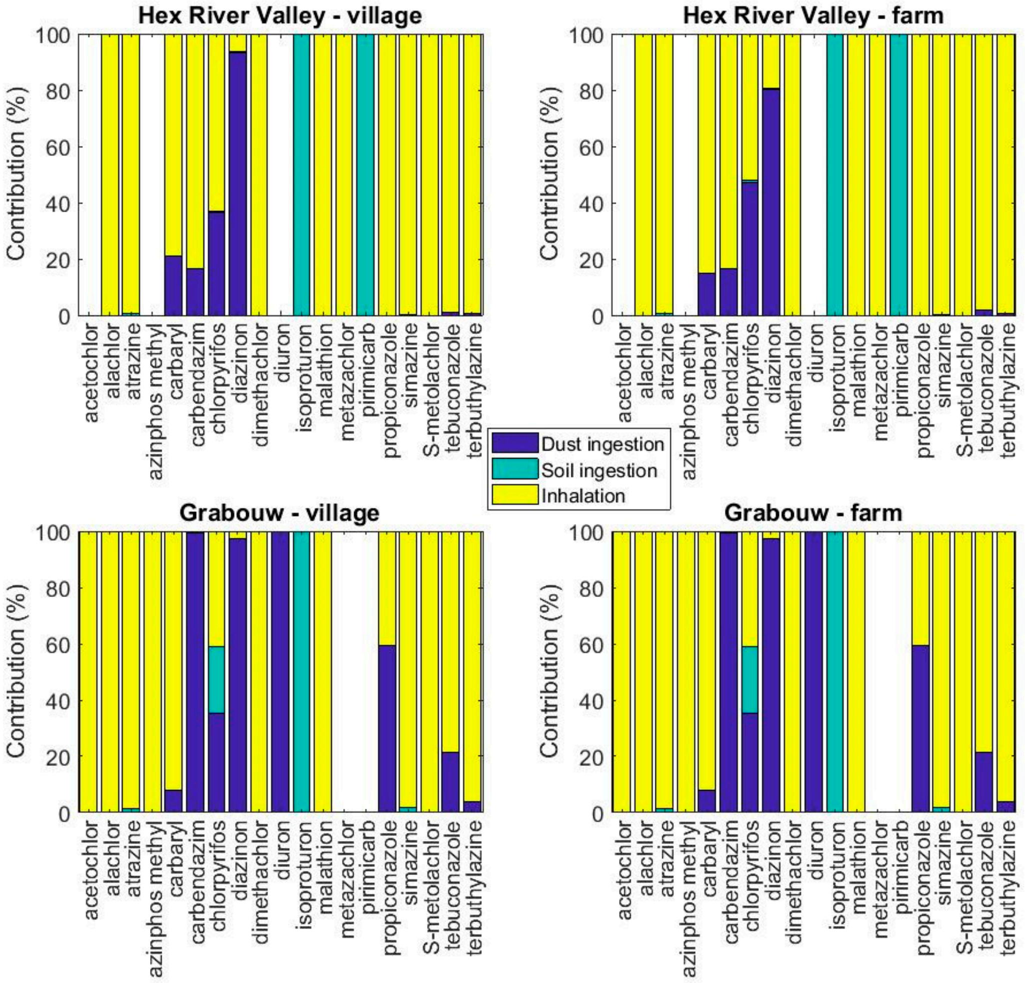

Figure S4: Contribution (in %) of three exposure pathways (dust ingestion, soil ingestion, and inhalation) on the daily uptake of pesticides of a children living at farm and village locations at Hex River Valley and Grabouw using the median concentrations and high ingestion rate. Data on the daily intakes from inhalation and soil ingestion of individual pesticides were obtained from [68].

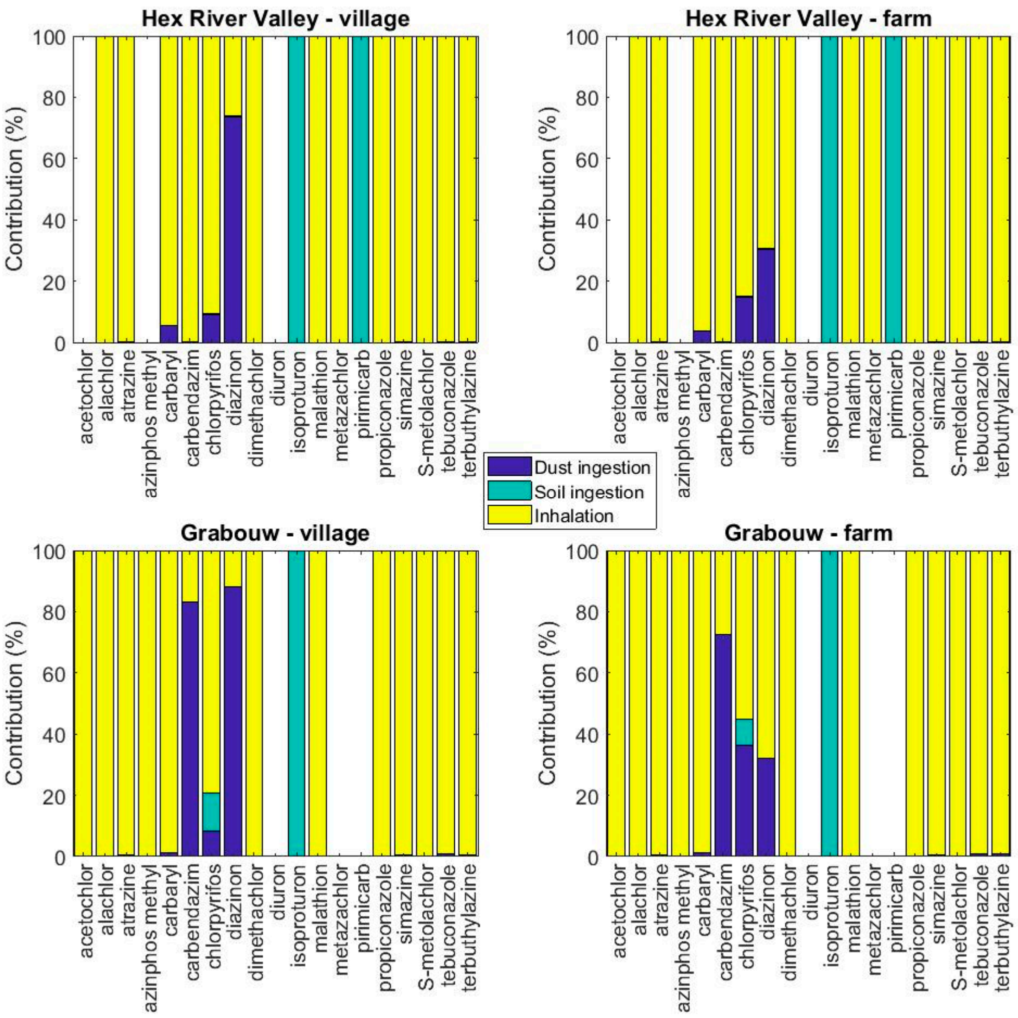

Figure S5: Contribution (in %) of three exposure pathways (dust ingestion, soil ingestion, and inhalation) on the daily uptake of pesticides of an adult living at farm and village locations at Hex River Valley and Grabouw using the median concentrations. Data on the daily intakes from inhalation and soil ingestion of individual pesticides were obtained from [68].

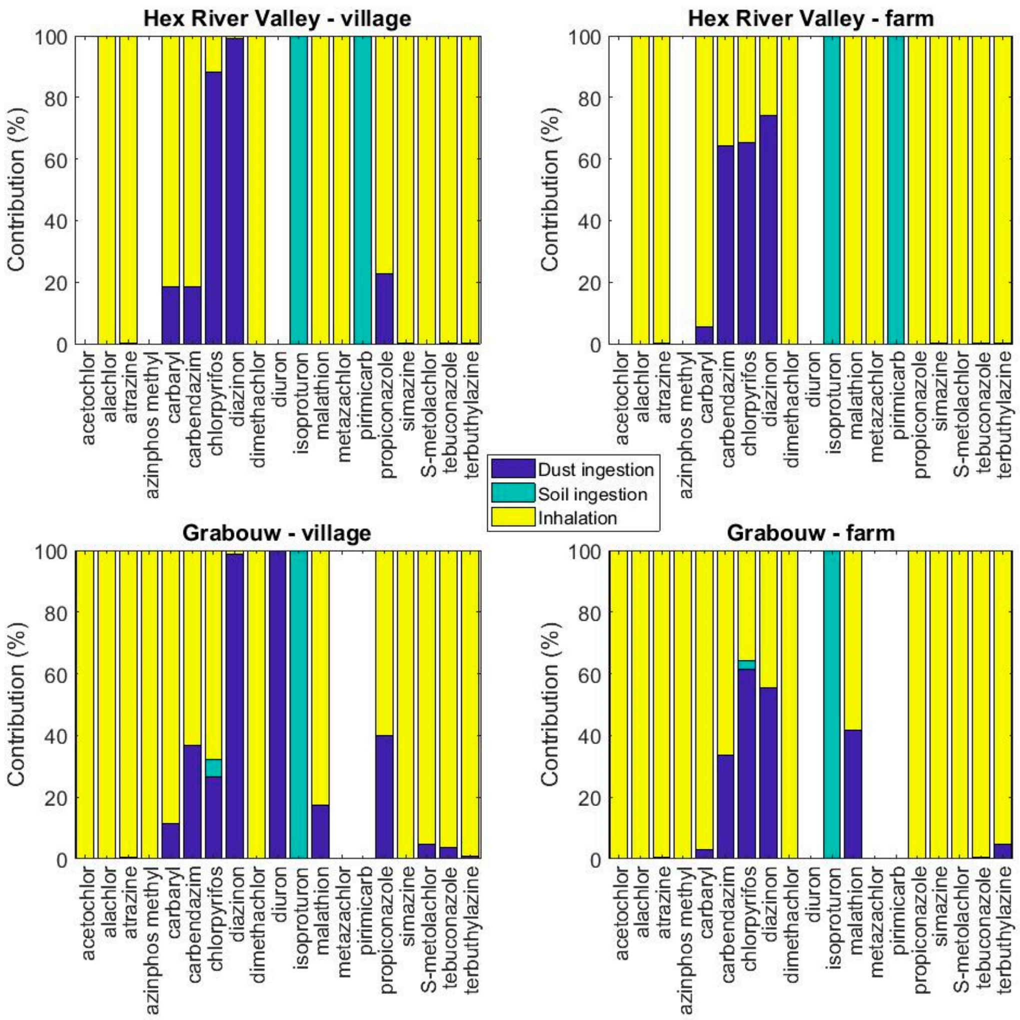

Figure S6: Contribution (in %) of three exposure pathways (dust ingestion, soil ingestion, and inhalation) on the daily uptake of pesticides of an adult living at farm and village locations at Hex River Valley and Grabouw using the maximum concentrations. Data on the daily intakes from inhalation and soil ingestion of individual pesticides were obtained from [68].

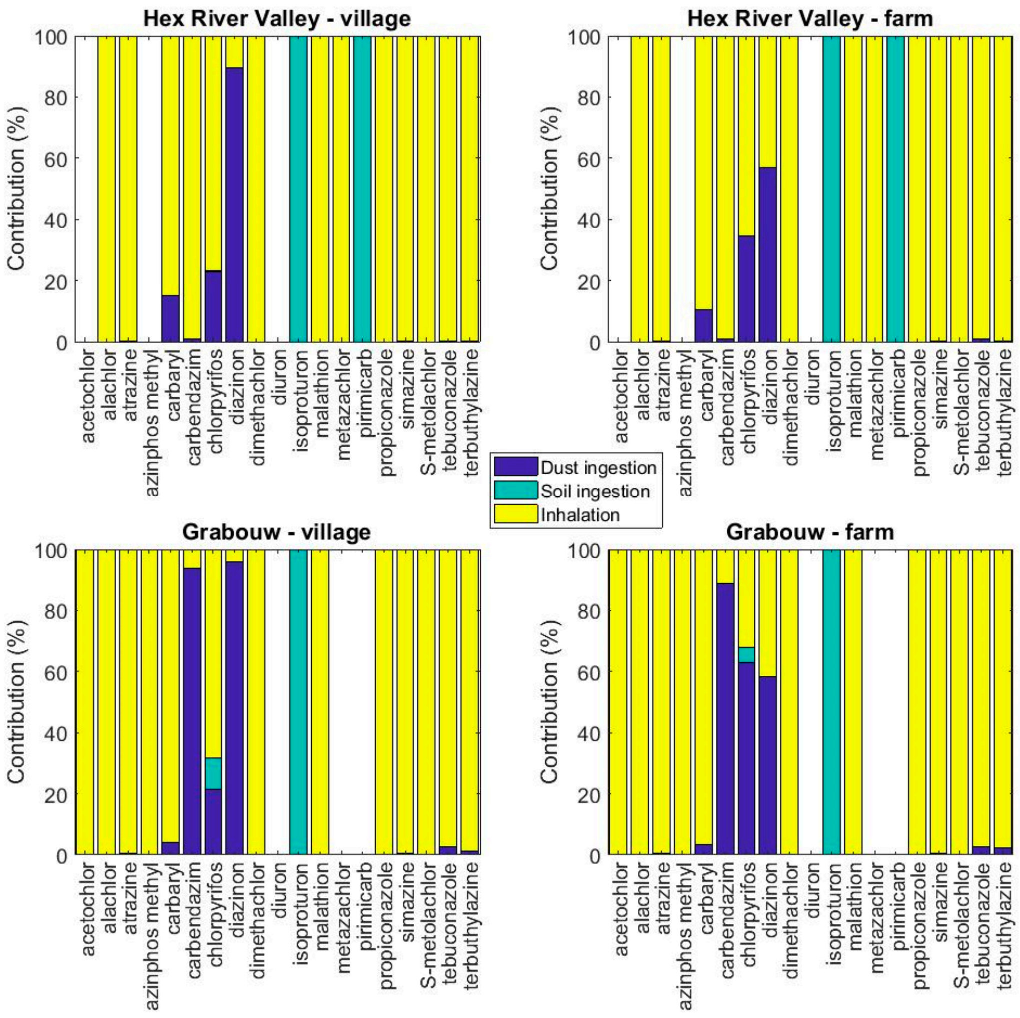

Figure S7: Contribution (in %) of three exposure pathways (dust ingestion, soil ingestion, and inhalation) on the daily uptake of pesticides of an adult living at farm and village locations at Hex River Valley and Grabouw using the median concentrations and the high ingestion rate of dust. Data on the daily intakes from inhalation and soil ingestion of individual pesticides were obtained from [68].
